# Associations Between a New York City Paid Sick Leave Mandate and Health Care Utilization Among Medicaid Beneficiaries in New York City and New York State

**DOI:** 10.1001/jamahealthforum.2021.0342

**Published:** 2021-05-06

**Authors:** Hansoo Ko, Sherry A. Glied

**Affiliations:** 1 New York University’s Robert F. Wagner Graduate School of Public Service, New York, New York

## Abstract

**Importance:**

More evidence on associations between mandated paid sick leave and health service utilization among low-income adults is needed to guide health policy and legislation nationwide.

**Objective:**

To evaluate the association between New York City’s 2014 paid sick leave mandate and health care utilization among Medicaid-enrolled adults.

**Design, Setting, and Participants:**

This retrospective cohort study used New York State Medicaid administrative data for adults 18 to 64 years old continuously enrolled in Medicaid from August 1, 2011, through July 31, 2017. A difference-in-differences approach with entropy balancing weights was used to compare New York City with the rest of New York State to assess the association of the paid sick leave mandate with health care utilization, and for those 40 to 64 years old, with preventive care utilization. The data analysis was performed from June through August 2020.

**Exposures:**

Temporal and spatial variation in exposure to the mandate.

**Main Outcomes and Measures:**

Annual health care utilization (emergency care, specialist visits, and primary care clinician visits) per Medicaid-enrolled adult. Secondary outcomes include categories of emergency utilization and utilization of 5 preventive services.

**Results:**

Of 552 857 individuals (mean [SD] age, 43 [12] years; 351 130 [64%] women) who met inclusion criteria, 99 181 (18%) were White, 162 492 (29%) Black, and 138 061 (25%) Hispanic. Paid sick leave was significantly associated with a reduction in the probability of emergency care (−0.6 percentage points [pp]; 95% CI, −0.7 to −0.5 pp; *P* < .001), including a 0.3 pp reduction (95% CI, −0.4 to −0.2; *P* < .001) in care for conditions treatable in a primary care setting and an increase in annual outpatient visits (0.124 pp; 95% CI, 0.040 to 0.208 pp; *P* < .001). Among those 40 to 64 years old, the mandate was significantly associated with increased probabilities of glycated hemoglobin A_1c_ level testing (2.9 pp; 95% CI, 2.5-3.3 pp; *P* < .001), blood cholesterol testing (2.7 pp; 95% CI, 2.5-2.9 pp; *P* < .001), and colon cancer screening (0.4 pp; 95% CI, 0.2-0.6 pp; *P* < .001).

**Conclusions and Relevance:**

This retrospective cohort study of nonelderly adults enrolled in Medicaid New York State showed that mandated paid sick leave in New York City was significantly associated with differences in several dimensions of health care services use.

## Key Points

**Question:**

What was the association between New York City's 2014 paid sick leave mandate and health care and preventive services use?

**Findings:**

This study of 552 857 nonelderly Medicaid beneficiaries in New York State from 2011 through 2017 found that the paid sick leave mandate was significantly associated with decreases in emergency department and specialist visits, increases in primary care use, and an increased probability of receiving certain preventive health services.

**Meaning:**

Mandated paid sick leave in New York City was significantly associated with small but important changes in health care and preventive services use among low-income adults.

## Teaser

This cohort study evaluates the association between New York City’s 2014 paid sick leave mandate and health care utilization among Medicaid-enrolled adults in New York City vs New York State.

## Introduction

Across the US, 29% of all workers and 68% of low-wage workers were ineligible for paid sick leave in 2019.^1^ A lack of paid sick leave has been posited as a reason for inappropriate emergency department utilization and for reduced use of preventive services.^2,3,4^ The enactment of a paid sick leave mandate in New York City offered an opportunity to assess the association of paid sick leave with emergency care and preventive health services utilization.

The US is 1 of only 2 member countries of the Organisation for Economic Co-operation and Development that do not mandate paid sick leave.^5^ There is growing interest in addressing this gap. During the COVID-19 pandemic, the US Congress has enacted a temporary national paid sick leave mandate. Prior studies^6^ have found that paid sick leave mandates increase the health care coverage rate without changing wage rates or employment levels. Sick leave mandates are also associated with lower population-level contagious respiratory disease rates,^7^ including lower rates of COVID-19,^8^ lower probabilities of working while sick,^9^ reduced occupational injuries and coronary events,^10^ and higher job retention rates among cancer patients.^11^ We hypothesized that mandating paid sick leave would lead to reductions in emergency department visits and specialist visits, increases in primary care visits, and increased preventive health care utilization.

## Methods

### Data and Study Sample

This retrospective cohort study followed the Strengthening the Reporting of Observational Studies in Epidemiology (STROBE) reporting guideline. The institutional review board of New York University reviewed and approved the analysis and waived the need for informed consent because deidentified data were used and all research materials were collected solely for administrative, nonresearch purposes.

Under New York City’s Paid Safe and Sick Leave Law,^12^ on April 1, 2014, full-time and part-time employees working in New York City became eligible for up to 40 hours of paid sick leave per year for the care and treatment of themselves or their family members. Paid sick days accrue at a rate of 1 hour per 30 hours of time worked and became available for use beginning on August 1, 2014. This law prohibits employers from requiring documentation that specifies the reason for paid sick leave or retaliating against employees for using sick leave.

Using complete New York State Medicaid deidentified claims data for August 1, 2011, through July 31, 2017, we constructed a balanced panel (eg, August 2011 through July 2012 as year 1) of adult Medicaid beneficiaries 18 to 64 years old who were continuously enrolled in Medicaid and did not change county of residence during this 6-year period. Those enrolled in Medicare (either 65 years or older or disabled) or aging into Medicare during the study period were excluded because these claims may have been incomplete in this study’s Medicaid-based data set. Also excluded were any records with incomplete demographic information.

### Outcomes

The primary outcomes studied were individual-level annual utilization of primary care clinicians, specialist physicians, and emergency departments, measured as any visit and as the number of visits within a year (12-month periods beginning in August from 2011 through 2017). We examined categories of emergency department utilization (emergency and not preventable, emergency and preventable, primary care–treatable, and nonemergency) as a secondary outcome, using an algorithm developed by Billings and colleagues,^2^ which assigns categories based on primary diagnosis (eAppendix 1 in the Supplement). We summed the probabilities of each visit type across all visit types for each individual to compute the annual number of emergency department visits classified by visit type.^13^ We used *Current Procedural Terminology* codes to identify annual use of 5 key preventive services: (1) glycated hemoglobin A_1c_ (HbA_1c_) testing, (2) blood cholesterol testing, and screenings for (3) colon, (4) breast, and (5) cervical cancer.

### Statistical Analysis

We used a difference-in-differences approach to compare secular (premandate vs postmandate) changes in health care utilization among New York City residents (the intervention group) with trends over the same time period among residents in the rest of New York State (the control group). We reported coefficients on the interaction term between the intervention group and the postpolicy period indicator (equal to 1 after August 2014; *β_1_* in the following equation) from linear multivariate models as follows:

Y*_it_* = *β_0_* + *β_1_* (*NYC_i_* × *Post_t_*) + *β_2_NYC_i_* + *β_3_Post_t_* + *α_i_* + *σ_t_* + *x_it_* + *ε_it_*

where *NYC* indicates a person *i* residing in New York City at the time *t* and *Post* denotes postmandate periods (year 4, year 5, and year 6, beginning with August 2014). The model controls for individual fixed effects (*α_i_*), which adjust for time-invariant unobserved individual factors, including baseline health status, health care preferences, race/ethnicity, and personality traits. To adjust for economic shocks common to the entire sample, regressions also included year fixed effects (*σ_t_*). We also included each individual’s Charlson Comorbidity Index score identified through primary diagnosis codes using the *International Classification of Diseases, Ninth Revision (ICD-9)* and *Tenth Revision (ICD-10)* as an individual-level time-variant factor (*x_it_*). For binary outcomes, we specified linear probability models to provide percentage-point (pp) changes in the probability of having any annual visit (eg, a coefficient estimate of 0.02 should be interpreted as a 2 pp increase in the probability of a visit).

To address differences in both observable and unobservable factors between the intervention group and the control group, we used entropy balancing. This technique reweights the control group based on the means and variances of sample covariates (age, gender, race/ethnicity, Charlson Comorbidity Index score, census tract median household income, and health care utilization in the premandate periods); this allowed creation of a balanced sample.^14^ We adjusted entropy weights to a standardized difference of 0.05, a commonly used threshold.^15^ We computed entropy balancing weights separately for the whole sample and for the older cohort 40 years and older (for preventive health service utilization analysis).^16^

Identification in the study model required the 2 groups to have had similar trends in outcomes in the absence of the mandate. To confirm that trends in the premandate period were not meaningfully different, we reported coefficients from an event study that allowed for the association of the mandate with outcome measures to vary over time:

Y*_it_* = α + ∑ ^2^*_j_*  _= −3_*β_j_* (*YearsSinceIntervention_j_* × *NYC_i_*) + *β_2_NYC_i_* + *Θ_i_* + *x_it_* + *ε_it_*

treating the year prior to the implementation of the policy as the omitted category; coefficients on year dummies can be interpreted as changes in utilization relative to this August 2013 to July 2014 reference period.^17^

To check the robustness of the study results, we also estimated difference-in-differences models without balancing weights and with nearest neighbor propensity score matching weights. We also tested the influence of including county-specific linear trends, allowing variation in outcome trends within substate administrative regions.^18^ In addition, we performed a placebo analysis as if the mandate had been enacted a year before its actual implementation.^19^

Under the mandate, workers accrue 1 hour of sick leave per 30 hours of time worked beginning on April 1, 2014. Thus, full-time workers had up to 2 full sick leave days available when the mandate was implemented on August 1, 2014, but none prior to that date. We conducted a sensitivity analysis using a regression discontinuity design applied to monthly data on New York City residents only (eAppendix 2 in the Supplement).

For the primary analysis, the unit of observation was the person-year. We reported standard errors clustered at the individual level and Bonferroni-corrected *P* values based on the Westfall-Young stepdown technique using a bootstrapping approach to adjust for multiple comparisons.^20^ *P* values were 2-tailed and statistical significance was defined as *P* < .05. Statistical analysis was performed from June through August 2020 using Stata, release 16.0 (StataCorp LLC).

## Results

### Baseline Characteristics

The study sample consisted of 552 857 adults (mean [SD] age, 43 [12] years; 351 130 [64%] women; 99 181 [18%] White, 162 492 [29%] Black, and 138 061 [25%] Hispanic) continuously enrolled in Medicaid (361 208 residing in New York City and 191 649 residing elsewhere in New York State) and totaled 3 317 142 person-year observations. For the preventive health services analysis, we focused on the population from 40 to 64 years old (n = 319 428) because of the greater number of preventive health services recommended for this age group.^21,22^ Table 1 reports summary statistics for the sample. Before matching, the intervention group had a higher mean age and a higher proportion of people of Hispanic or other race/ethnicity (non-White, non-Black, or non-Hispanic), a lower number of annual emergency department visits, and a higher Charlson Comorbidity Index score. After reweighting with entropy balancing, the standardized difference of means of all the variables fell below the specified threshold of *P* < .05.^15^

**Table 1.** Descriptive Statistics of Variables and Matching Balancing Properties at Baseline of 552 857 Adults 18-64 Years Old Continuously Enrolled in New York State Medicaid From August 2011 Through July 2017

Variable	Before matching			After matching^a^		
	New York City (paid sick leave)	New York State (no paid sick leave)	Standardized difference	New York City (paid sick leave)	New York State (no paid sick leave)	Standardized difference
No. of persons	361 208	191 649	NA	361 208	191 649	NA
Mean age, y	44.00	40.86	0.271	44.00	44.00	<0.001
Median, y (IQR)	45 (35-54)	40 (31-51)	NA	45 (35-54)	45 (34-54)	NA
Sex						
Female	0.63	0.65	−0.061	0.623	0.623	<0.001
Male	0.37	0.35	−0.061	0.38	0.38	<0.001
Race/ethnicity^b^						
Asian/other/unknown	0.34	0.17	0.391	0.34	0.34	<0.001
Hispanic	0.32	0.13	0.475	0.32	0.32	<0.001
Non-Hispanic						
White	0.18	0.18	−0.009	0.18	0.18	<0.001
Black	0.17	0.26	−0.802	0.17	0.17	<0.001
Charlson Comorbidity Index score^c^	0.58	0.39	0.162	0.58	0.58	<0.001
Any annual primary care clinician visit	0.78	0.79	−0.010	0.78	0.78	<0.001
No. of annual primary care clinician visits	5.05	4.63	0.048	5.05	0.59	<0.001
Any annual specialist visit	0.59	0.62	−0.061	0.59	0.59	<0.001
No. of annual specialist visits	3.90	4.05	−0.015	3.90	3.90	<0.001
Any ED visit	0.45	0.59	−0.289	0.45	0.45	−0.006
No. of annual ED visits	0.75	1.11	−0.191	0.75	0.75	<0.001
ED visit^d^						
Not preventable	0.10	0.13	−0.054	0.10	0.10	<0.001
Preventable	0.05	0.07	−0.056	0.05	0.05	<0.001
Primary care–treatable	0.19	0.26	−0.139	0.19	0.19	<0.001
Nonemergency	0.22	0.29	−0.111	0.22	0.22	<0.001
Census tract median household income, $	50 519	60 288	−0.351	50 519	50 519	<0.001
Census tract poverty rate	0.18	0.11	0.557	0.18	0.18	<0.001

Abbreviations: ED, emergency department; IQR, interquartile range; NA, not applicable.

^a^ Means and variances of the control group sample were reweighted with entropy weights for covariates listed above to be balanced to a standardized difference of 0.05.

^b^ Race/ethnicity was self-identified when applicants enrolled in Medicaid based on fixed categories (Black, White, Hispanic, and [combined] Asian, other, or unknown) defined by Medicaid. We classified race/ethnicity to these mutually exclusive categories.

^c^ The score is the sum of assigned scores (depending on the predicted risks of 1-year mortality) for 22 chronic conditions (higher scores indicate higher predicted mortality risks; score ranges from 0-37). We calculated each person’s score within a given year. Among the total 3 317 142 person-year observations, 29% had scores >0 and the highest score was 21.

^d^ Using Billings and colleagues’ algorithm^2^ that assigns the likelihood that a visit is of a particular type based on primary diagnosis, the annual number of ED visits was classified by summing all the probabilities of being each type across all visits for a person. These categories are, as recommended, not mutually exclusive (details available in eAppendix 1 in the Supplement).

### Association of Mandated Paid Sick Leave With Utilization

Table 2 describes the association of the New York City paid sick leave mandate with the study’s primary outcome measures. Mandated paid sick leave was significantly associated with 4 of the 6 prespecified outcomes. Average emergency and specialist visits in this continuously enrolled cohort increased in the rest of New York State, but did not increase in New York City. Thus, the mandate was significantly associated with a reduction in emergency department visits; fewer people had any visits within a given year (−0.6 pp; 95% CI, −0.7 to −0.5 pp; *P* < .001) and per-person annual visits declined (−0.022 pp; 95% CI, −0.026 to −0.018 pp; *P* < .001). Relative to the premandate period means, these estimates imply a 1.2% reduction in the probability of any annual visit and a 2.5% reduction in the number of annual visits. The mandate was also significantly associated with a 1.1 pp reduction in the probability of a specialist physician visit (95% CI, −0.013 to −0.009 pp; *P* < .001), a 1.8% relative reduction.

**Table 2.** Associations of the 2014 New York City Paid Sick Leave Mandate With Annual Health Care Utilization Before and After Mandate Implementation: Difference-in-Differences With Entropy-Balancing Weights

Utilization type^a^	Mean (SD)^b^				Difference-in-differences estimates (95% CI)^c^	Bonferroni-corrected *P* value	Relative change compared with premandate mean, %
	New York City residents (n = 361 208)		New York State residents (n = 191 649)				
	Before	After	Before	After			
Any annual ED visit	0.447 (0.497)	0.447 (0.498)	0.447 (0.497)	0.454 (0.498)	−0.006 (−0.007 to −0.005)	<.001	−1.21
No. of annual ED visits	0.752 (1.679)	0.752 (1.680)	0.752 (1.330)	0.774 (1.441)	−0.022 (−0.026 to −0.018)	<.001	−2.52
Any annual specialist visit	0.594 (0.491)	0.595 (0.491)	0.594 (0.491)	0.606 (0.489)	−0.011 (−0.013 to −0.009)	<.001	−1.82
No. of annual specialist visits	3.909 (10.176)	3.895 (10.040)	3.909 (9.181)	3.895 (10.040)	−0.021 (−0.062 to 0.020)	.63	−0.53
Any annual primary care clinician visit	0.783 (0.412)	0.783 (0.411)	0.783 (0.411)	0.782 (0.413)	0.0002 (−0.002 to 0.004)	.81	0.03
No. of annual primary care clinician visits	5.039 (10.050)	5.044 (10.073)	5.039 (10.155)	4.925 (9.379)	0.124 (0.040 to 0.208)	.01	2.53

Abbreviation: ED, emergency department.

^a^ Primary care clinician (not including specialist physician) visits, specialist physician visits, and ED visits were identified based on Medicaid’s billing classification.

^b^ Means and variances were reweighted with entropy balancing weights.

^c^ These adjusted coefficient estimates were estimated from linear regressions (for continuous variables) or linear probability regressions (for binary variables) that controlled for year dummies, individual fixed effects, and Charlson Comorbidity Index scores. Standard errors are clustered at the individual level.

Average primary care visits declined in the rest of New York State, but not in New York City, thus the mandate was also significantly associated with an increase in the number of annual outpatient clinician visits (0.124 pp; 95% CI, 0.040-0.208 pp; *P* < .001), equivalent to a 2.5% change relative to the premandate period mean. The mandate was associated with a reduced number of annual specialist physician visits (−0.021 pp; 95% CI, −0.062 to 0.020 pp; *P* = .63) and an increased probability of any primary care clinician visit (0.02 pp; 95% CI, −0.2 to 0.4 pp; *P* = .81), but these estimates were not statistically significant.

The Figure reports event study estimates (coefficient estimates and 95% CIs are reported in eTable 1 in the Supplement). The negative associations of the mandate with emergency department utilization, both in whether a visit occurred and in the number of annual visits, and with the probability of any specialist physician visit were statistically significant during the year after the mandate was implemented and persisted thereafter. The mandate was significantly associated with an increased number of annual primary care clinician visits among beneficiaries in the years after the mandate. There was no evidence of differential trends in outcomes between the intervention group and the control group in the premandate period (year −3 and year −2).

**Figure.**
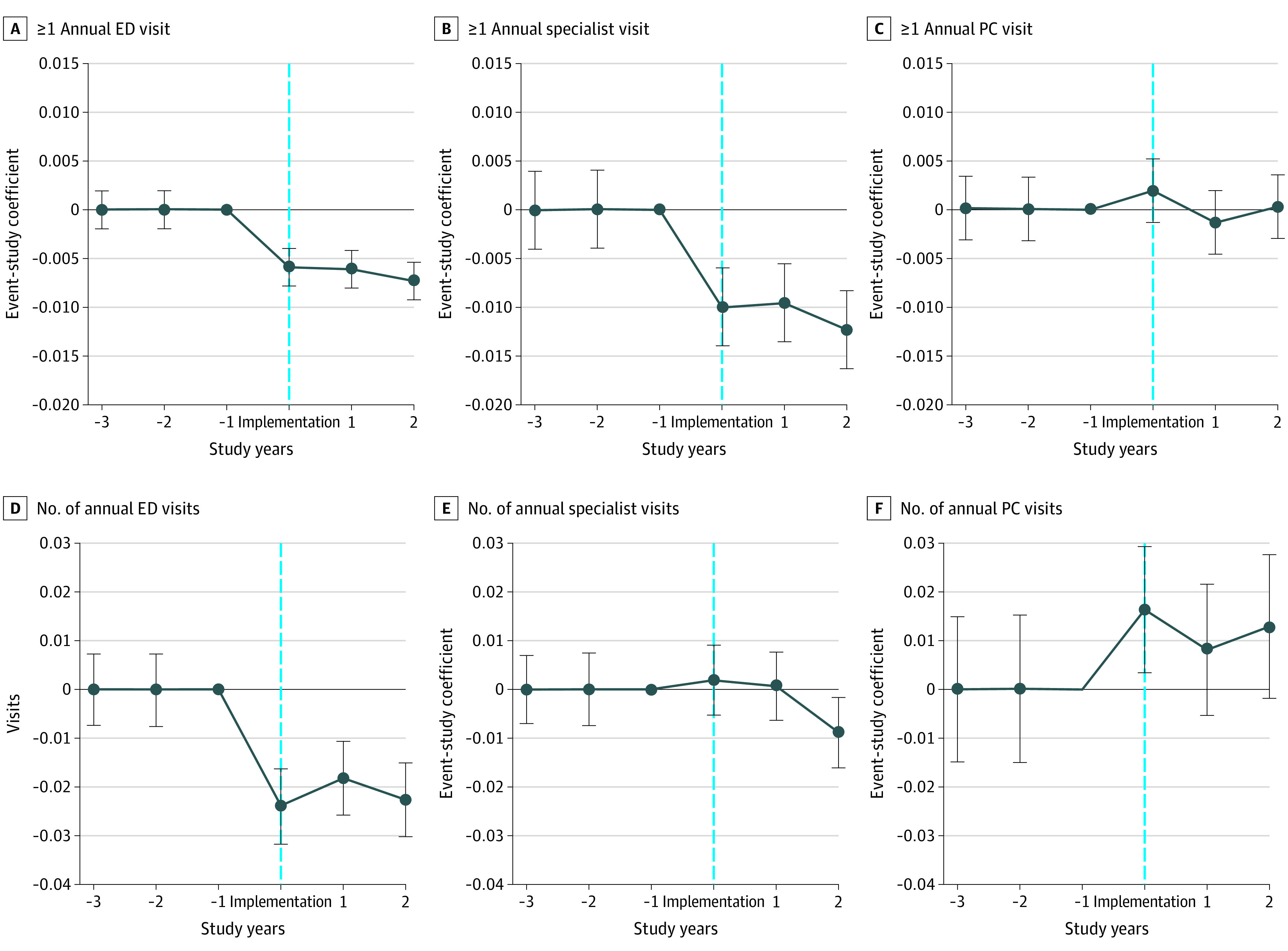
Association of the 2014 New York City Paid Sick Leave Mandate With Annual Health Care Utilization: Event Study Abbreviations: ED, emergency department; PC, primary care clinician. Dots represent event-study coefficient estimates. Vertical bars represent 95% CIs (eTable 1 and eTable 2 in the Supplement) from the equation: Y*_it_* = α + ∑ ^2^*_j_* _= −3_*β_j_* (*YearsSinceIntervention_j_* × *NYC_i_*) + *β_2_NYC_i_* + *Θ_i_* + *x_it_* + *ε_it_* where NYC represents a person *i* who at the time *t* was residing in New York City; *Θ_i_* denotes individual fixed effects and time-varying factor; and *x_it_* is the Charlson Comorbidity Index score. Category omitted: the year prior to mandate implementation (August 1, 2013-July 31, 2014). Regressions used sample reweighted by entropy balancing.

In Table 3, we report associations between the mandate and categories of emergency care. The association between the mandate and emergency department visits classified as primary care–treatable was significantly negative, reflecting a reduction in these visits by 1.4% compared with the premandate period mean (−0.003 visits; 95% CI, −0.004 to −0.002 visits; *P* < .001). This association appeared immediately when the mandated paid sick leave was implemented and persisted thereafter (Figure; eTable 2 in the Supplement). We did not find significant associations between the mandate and other types of emergency care: emergency and not preventable (0.0002 visits; 95% CI, −0.001 to 0.001 visits), emergency and preventable (−0.0008 visits; 95% CI, −0.002 to 0.00002 visits), and nonemergency (−0.002 visits; 95% CI, −0.004 to 0.00004 visits).

**Table 3.** Associations of the 2014 New York City Paid Sick Leave Mandate With Emergency Care Utilization by Visit Type Before and After Mandate Implementation: Difference-in-Differences With Entropy-Balancing Weights

Visit type^a^	Mean (SD)^b^				Difference-in-differences estimates (95% CI)^c^	Bonferroni-corrected *P* value	Relative change compared with the premandate mean, %
	New York City residents (n = 361 208)		New York State residents (n = 191 649)				
	Before	After	Before	After			
Emergency							
Not preventable	0.101 (0.534)	0.101 (0.559)	0.101 (0.551)	0.099 (0.531)	0.0002 (−0.001 to 0.001)	.80	0.18
Preventable	0.054 (0.325)	0.054 (0.320)	0.054 (0.349)	0.054 (0.347)	−0.0008 (−0.002 to 0.00002)	.18	−1.36
Primary care–treatable	0.185 (0.491)	0.182 (0.502)	0.185 (0.480)	0.184 (0.481)	−0.003 (−0.004 to −0.002)	<.001	−1.42
Nonemergency	0.219 (0.566)	0.216 (0.577)	0.219 (0.574)	0.218 (0.577)	−0.002 (−0.004 to 0.00004)	.23	−0.82

^a^ Emergency department utilization was classified by using Billings and colleagues’ algorithm^2^ that assigns the likelihood that a visit is of a particular type on the basis of primary diagnosis. The annual number of emergency department visits classified by visit type is obtained by summing all the probabilities of being each type across all visits for a person (see eAppendix 1 in the Supplement for details).

^b^ Means and variances were reweighted with entropy balancing weights.

^c^ These adjusted coefficient estimates were estimated from linear regressions (for continuous variables) or linear probability regressions (for binary variables) that controlled for year dummies, individual fixed effects, and Charlson Comorbidity Index scores. Standard errors are clustered at the individual level.

Table 4 shows estimated associations of paid sick leave with preventive service utilization among adult Medicaid beneficiaries from 40 to 64 years old. The mandate was significantly associated with a 2.9 pp increase in the probability of an HbA_1c_ level test within a given year (5.3% relative increase from the premandate mean; 95% CI, 2.5-3.3 pp; *P* < .001), a 2.7 pp increase in the probability of receiving a blood cholesterol test (a 3.6% relative increase; 95% CI, 2.5-2.9 pp; *P* < .001), and a 0.4 pp increase in the probability of receiving a colon cancer screening (a 2.4% relative increase; 95% CI, 0.2-0.6 pp; *P* < .001). The associations between the mandate and breast cancer screening (−0.4 pp; 95% CI, −0.8 to 0.1 pp) or cervical cancer screening (−0.06 pp; 95% CI, −0.5 to 0.3 pp) among women enrolled in Medicaid were not statistically significant. There was no evidence of differential trends in these outcomes prior to mandate implementation (eFigure 1 and eTable 3 in the Supplement), but coefficients on associations between the mandate and receiving an HbA_1c_ test, a blood cholesterol test, and a colon cancer screening rose significantly after implementation of the mandate.

**Table 4.** Associations of the 2014 New York City Paid Sick Leave Mandate With Adult (40-64 years old) Preventive Care Utilization Before and After Mandate Implementation: Difference-in-Differences With Entropy-Balancing Weights

Preventive services^a^	Mean (SD)^b^				Difference-in-differences estimates (95% CI)^c^	Bonferroni-corrected *P* value	Relative change compared with the premandate mean, %
	New York City residents (n = 361 208)		New York State residents (n = 191 649)				
	Before	After	Before	After			
Any annual test							
Glycated hemoglobin A_1c_	0.598 (0.490)	0.590 (0.492)	0.596 (0.499)	0.574 (0.490)	0.029 (0.025 to 0.033)	<.001	5.28
Cholesterol	0.776 (0.417)	0.769 (0.421)	0.782 (0.415)	0.749 (0.419)	0.027 (0.025 to 0.029)	<.001	3.64
Any annual cancer screening							
Colon	0.168 (0.374)	0.157 (0.364)	0.167 (0.367)	0.152 (0.356)	0.004 (0.002 to 0.006)	<.001	2.42
Breast^d^	0.541 (0.498)	0.534 (0.499)	0.537 (0.489)	0.533 (0.485)	−0.004 (−0.008 to 0.001)	.38	−0.76
Cervical^d^	0.279 (0.448)	0.290 (0.454)	0.276 (0.438)	0.289 (0.436)	−0.0006 (−0.005 to 0.003)	.91	−0.23

^a^ Outcomes measure the probability of receiving indicated preventive service per person within a given year.

^b^ Means and variances were reweighted with entropy balancing weights.

^c^ These adjusted coefficient estimates were estimated from linear regressions (for continuous variables) or linear probability regressions (for binary variables) that controlled for year dummies, individual fixed effects, and Charlson Comorbidity Index scores. Standard errors are clustered at the individual level.

^d^ Among female Medicaid beneficiaries (n = 194 186).

### Sensitivity Checks

These results were robust to the sensitivity tests described (eTable 4 in the Supplement), although the estimated association of the mandate with emergency care utilization using a traditional difference-in-differences approach and the estimated association between the mandate and primary care clinician visits in the model including linear trends became statistically insignificant. Results from a placebo regression showed no association of the mandate with outcome measures before its actual implementation.

Results from the monthly regression discontinuity design analysis (eTable 5 and eFigure 2 in the Supplement) showed that mandated paid sick leave was associated with significant changes at month 0 (August 2014), indicating fewer monthly emergency department visits (−0.0016 pp; 95% CI, −0.0010 to −0.0021 pp; *P* < .001), more primary care clinician visits (0.013 pp; 95% CI, 0.010 to 0.015 pp; *P* < .001), and reduced emergency care utilization for conditions treatable in primary care settings (−0.0004 pp; 95% CI, −0.0006 to −0.0003 pp; *P* < .001). Compared with the premandate period mean, the mandate was associated with a 5.9% reduction in the number of monthly emergency department visits, a 6.7% reduction in emergency care utilization for conditions treatable in a primary care setting, and a 3.3% increase in the number of monthly primary care clinician visits. We did not find that the mandate was significantly associated with monthly changes in emergency department visits for conditions not preventable, preventable, or nonemergency. Implementation of the mandate was associated with an increasingly positive trend in primary care utilization (0.002 pp; 95% CI, 0.002-0.003 pp; *P* < .001).

## Discussion

Reducing use of the emergency department for primary care–treatable conditions has long been a US policy priority.^2^ We found that New York City’s 2014 Paid Safe and Sick Leave Law was associated with a small but significant shift in health care utilization among Medicaid-enrolled nonelderly adults, away from emergency and specialty care and toward primary care clinician visits and preventive health services. Emergency care increased among nonelderly Medicaid beneficiaries in New York State (outside of New York City), consistent with literature reporting increasing trends in emergency department use nationwide from 2005 through 2016,^23^ and with the aging of the continuously enrolled cohort, but emergency department use did not increase in New York City after the paid sick leave mandate was implemented. The mandate was associated with a significant (2.5%) reduction in annual emergency department visits, equal to 8000 fewer emergency department visits by adult, nonelderly Medicaid beneficiaries in New York City annually.^24^

Implementation of the mandate was associated with a positive trend in monthly primary care clinician visits, consistent with a gradual increase in utilization as workers accrued more paid sick leave days. Mandated paid sick leave was also significantly associated with increases of 2% to 5% in the probability that low-income adults 40 to 64 years old received preventive services (HbA_1c_ test, cholesterol test, and colon cancer screening). These findings of a contemporaneous increase in primary care utilization, a reduction in specialty visits, and increases in preventive service utilization associated with paid sick leave are consistent with prior research.^25,26^

### Limitations

This study has several limitations. First, these study results are specific to the continuously enrolled New York State Medicaid population. We focused on continuously enrolled beneficiaries to avoid confounding the effects of the mandate with those of the Medicaid expansion of 2014 under the Affordable Care Act. The continuously enrolled population was proportionately younger, more female, and more Hispanic than the total Medicaid adult population; however, it may also have had greater medical needs than other beneficiaries. To assess how sample restriction may have affected the findings, we repeated the difference-in-differences analysis (without entropy weights) using a 10% sample of all nonelderly, nondual (not also enrolled in Medicare) Medicaid-enrolled adults. The association of the mandate with the probability of any annual emergency department use in this sample was statistically significant and slightly greater (−0.6 pp) than in the primary study sample (−0.5 pp); patterns of association with specialty visits (−1.1 pp) and primary care visits (0.6 pp) were also similar.

Second, sick leave is only relevant for workers, and we do not know the employment status of the study population. Because the paid sick leave mandate also applied to part-time workers and the New York Medicaid income-eligibility limit in 2013 was relatively high (100% of the Federal poverty level for single adults; 150% for parents), a substantial share of the study sample would have been eligible for benefits. Using the 2018 American Community Survey,^27^ we found that 53.5% of all adults enrolled in Medicaid in New York City had worked during the prior week. This estimate suggests that the mandate’s influence on working Medicaid beneficiaries may have been as much as twice as high because some may have benefited from a family member’s paid sick leave benefits; this is an upper bound.

Third, substitution of primary care for emergency care may be more likely among those with working hours that coincide with clinicians’ hours. The US Bureau of Labor Statistics reported^28^ that about one-fifth of low-wage workers have nontraditional work schedules. Fourth, the Billings and colleagues algorithm^2^ used to categorize emergency department visits may overstate the proportion that were primary care–treatable. Fifth, we found that the mandate was significantly associated with colon cancer screening, but its associations with breast cancer and cervical cancer screenings were not significant. Further research is needed to understand differential determinants of cancer screening in among people of low income.

## Conclusions

In this cohort study of New York State Medicaid administrative data for adults, we found that New York City's 2014 paid sick leave mandate was significantly associated with decreases in emergency care and in specialist physician visits and with increases in outpatient primary care clinician visits and the probability of receiving some preventive health services compared with the rest of New York State. While a 2.5% reduction is modest in the context of emergency department use overall, it is substantial by contrast to the null effects observed in studies of other strategies (eg, cost-sharing)^29,30^ that aim to reduce emergency department use among people enrolled in Medicaid. The reduction, especially in visits for conditions that are primary care–treatable, is also clinically important because overcrowded emergency departments can reduce the capacity of health care professionals to provide appropriate resources to patients with critical conditions. Further research among other populations is needed to understand the generalizability of these findings,^9^ especially now that the US Congress and several other states and cities^31^ are considering or have passed mandates to make paid sick leave a permanent benefit.

## Notes

Supplement 1. **eAppendix 1.** Classification of emergency department visits. **eAppendix 2.** Regression discontinuity design. **eFigure 1.** Associations of the 2014 New York City paid sick mandate with adults’ (40-64 years old) preventive care utilization: event study **eFigure 2.** Changes in monthly utilization rates before and after implementation (August 2014) of paid sick leave mandate among adult (18-64 years old) Medicaid beneficiaries in New York City. **eTable 1.** Associations of the 2014 New York City paid sick mandate with annual health care use: event study. **eTable 2.** Associations of the 2014 New York City paid sick mandate with emergency care utilization by type of visit: event study. **eTable 3.** Associations of the 2014 New York City paid sick mandate with adults’ (40-64 years old) preventive care utilization: event study. **eTable 4.** Associations of the 2014 New York City paid sick mandate with annual health care utilization: alternative specification. **eTable 5.** Changes in monthly utilization rates: regression discontinuity design.
