# Supplementary material for: Associations Between a New York City Paid Sick Leave Mandate and Health Care Utilization Among Medicaid Beneficiaries in New York City and New York State
Source: JAMA Health Forum. 2021 May 6;2(5):e210342. doi: 10.1001/jamahealthforum.2021.0342 (PMC8796973; doi:10.1001/jamahealthforum.2021.0342)
Supplement: Supplement 1. — eAppendix 1. Classification of emergency department visits. eAppendix 2. Regression discontinuity design. eFigure 1. Associations of the 2014 New York City paid sick mandate with adults’ (40-64 years old) preventive care utilization: event study eFigure 2. Changes in monthly utilization rates before and after implementation (August 2014) of paid sick leave mandate among adult (18-64 years old) Medicaid beneficiaries in New York City. eTable 1. Associations of the 2014 New York City paid sick mandate with annual health care use: event study. eTable 2. Associations of the 2014 New York City paid sick mandate with emergency care utilization by type of visit: event study. eTable 3. Associations of the 2014 New York City paid sick mandate with adults’ (40-64 years old) preventive care utilization: event study. eTable 4. Associations of the 2014 New York City paid sick mandate with annual health care utilization: alternative specification. eTable 5. Changes in monthly utilization rates: regression discontinuity design. [file jamahealthforum-e210342-s001.pdf]

Supplemental Online Content

Ko H, Glied SA. Associations between a New York City paid sick leave mandate and health care utilization among Medicaid beneficiaries in New York City and New York State. *JAMA Health Forum*. 2021;2(5):e210342. doi:10.1001/jamahealthforum.2021.0342

eAppendix 1. Classification of emergency department visits.

eAppendix 2. Regression discontinuity design.

eFigure 1. Associations of the 2014 New York City paid sick mandate with adults' (40-64 years old) preventive care utilization: event study

eFigure 2. Changes in monthly utilization rates before and after implementation (August 2014) of paid sick leave mandate among adult (18-64 years old) Medicaid beneficiaries in New York City.

eTable 1. Associations of the 2014 New York City paid sick mandate with annual health care use: event study.

eTable 2. Associations of the 2014 New York City paid sick mandate with emergent care utilization by type of visit: event study.

eTable 3. Associations of the 2014 New York City paid sick mandate with adults' (40-64 years old) preventive care utilization: event study.

eTable 4. Associations of the 2014 New York City paid sick mandate with annual health care utilization: alternative specification.

eTable 5. Changes in monthly utilization rates: regression discontinuity design.

This supplemental material has been provided by the authors to give readers additional information about their work.

© 2021 Glied SA et al. *JAMA Health Forum.*

eAppendix1. Classification of emergency department visits.

We used categorized emergency department utilization by using Billings et al.'s algorithm,[1] which assigned likelihoods to each visit being each type (emergent and not preventable, emergent and preventable, primary care treatable, and non-emergent) based on primary discharge diagnosis. The algorithm was developed by a panel of emergency department physicians to map of initial complaints to discharge diagnoses on complete 3,500 emergency care records (containing information on initial chief complaints, vital signs, patient's demographics and medical history, procedures performed in emergency departments, and discharge diagnoses) from New York-area hospitals. For each primary discharge diagnosis, the algorithm obtained the probabilities of each type of visit by averaging all the physicians' ratings. Because of this logic, common conditions may contribute to multiple categories. For instance, among emergency department visits with a discharge ICD-9 code "005.9" (food poisoning), 17% were classified as not preventable and emergent, 46% were emergent but primary care-treatable, and 37% were not emergent. Following a previous study on access to care among Medicaid enrollees,[2] we obtained the annual number of emergency department visits classified by type of visit by summing all the probabilities of each type of visit across all visits for a person within a given year.

© 2021 Glied SA et al. *JAMA Health Forum.*

eAppendix2. Regression discontinuity design.

The key assumption of our analyses is that the effects should be concentrated in the second half of 2014 because accumulation of sick leave hours began in April 2014 but paid sick leave became available to use beginning in August 2014. In other words, our utilization measures would have followed the same trends without the policy and we would find smooth trend lines at the cutoff date. To explore whether there were discrete changes in utilization when paid sick leave was implemented in New York City in August 2014, we specify a regression discontinuity model as follows:

(1) $Y_{it} = \alpha + \beta_1 X_t + \beta_2 \delta t X_t + \theta_i + \beta_0 t + \varepsilon_{it}$

Unit of analysis is person-month. Our outcome measure Y_{it} is monthly utilization per person including primary care clinician visit and emergency room visit. In particular, classifying emergent utilization into the four types of visit,[1] we expect to find significant reductions in emergency room utilization for conditions classified as primary care treatable and insignificant changes in not-preventable emergent utilization. A binary indicator X_t equals 1 in and after August 2014 (t ≥0, t ranges from -12 to 11) and a parameter β_1 shows discontinuous changes in outcome measures at month zero attributed to the implementation of paid sick leave – for our best knowledge, no other changes in Medicaid utilization policy were concurrently made in August 2014. δt is slopes of linear trend and a parameter β_2 represents a gradual change in trends (the difference in the slopes between the pre-policy period and the post-policy period). We are interested in the statistical significance and direction of coefficient estimates on β_1 and β_2. For instance, if paid sick leave led to immediate reductions in emergency room utilization in August 2014, estimated β_1 would be negative and statistically significant. Rather, if the policy resulted in a gradual reduction after the implementation, we would see significantly negative β_2.

Unlike a previous study relying on a regression discontinuity framework,[3] our strength is to

© 2021 Glied SA et al. *JAMA Health Forum.*

have detailed information on outcome measures at the individual level. Including individual fixed effects (θ_i) in the regression model, we are able to adjust for unobservable time-invariant individual characteristics that may confound the association between paid sick leave and healthcare utilization. A vector of calendar month dummies (Z_t ; February through December) is also included for controlling for seasonality. Standard errors are clustered at the individual level.

For graphical presentations shown in eFigure2, we first estimate seasonality-adjusted monthly utilization rate using the following regression:

(2) $Y_{it} = \alpha + \gamma Z_t + \theta_i + \varepsilon_{it}$

Taking regression coefficients on calendar month dummies ($\hat{\gamma}$), we calculate seasonality-adjusted rates ($\overline{Y_t}$-$\hat{\gamma}Z_t$) and present the results eFigure 2.[3]

© 2021 Glied SA et al. *JAMA Health Forum.*

eReferences

1. Billings J, Parikh N, Mijanovich T. Emergency room use: The New York Story. https://www.commonwealthfund.org/publications/issue-briefs/2000/nov/emergency-room-use-new-york-story. Accessed December 27, 2020.

2. Taubman SL, Allen HL, Wright BJ, Baicker K, Finkelstein AN. Medicaid increases emergency-department use: Evidence from Oregon's Health Insurance Experiment. *Science*. 2014; 343:263-268.

3. Nazzal C, Harris JE. 2017. Lower incidence of myocardial infarction after smoke-free legislation enforcement in Chile. *Bulletin of the World Health Organization* 95: 674-682.

© 2021 Glied SA et al. *JAMA Health Forum.*

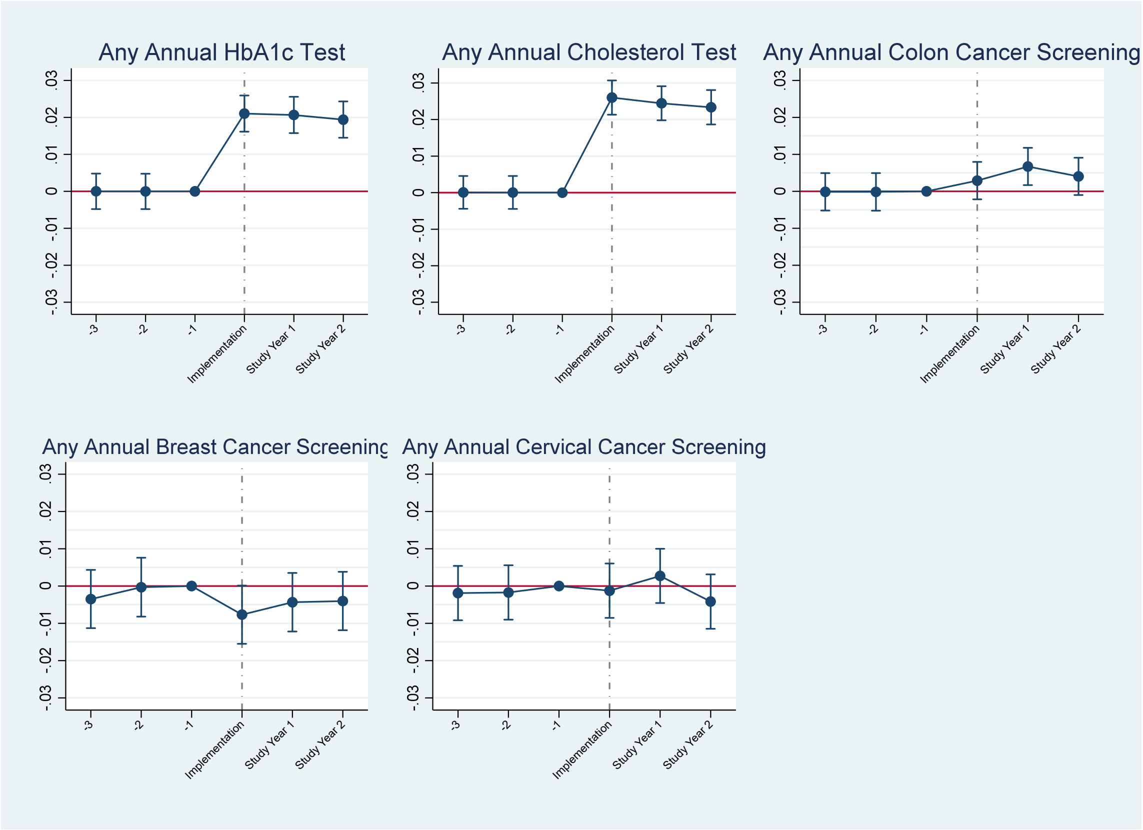

eFigure 1. Associations of the 2014 New York City paid sick mandate with adults' (40-64 years old) preventive care utilization: event study

Study sample included continuously-enrolled Medicaid beneficiaries aged 40–64 in New York State between August 2011– July 2017. Regression coefficients and 95% confidence interval (eTable 3) were taken from regressions of outcome measures on the interactions between New York City indicator and year dummies (omitted category; the year prior to the implementation (August 2013–July 2014)). Regressions used sample reweighted by entropy balancing and included individual fixed effects, year dummies, and Charlson comorbidity index. Standard errors were clustered at the individual level.

© 2021 Glied SA et al. *JAMA Health Forum.*

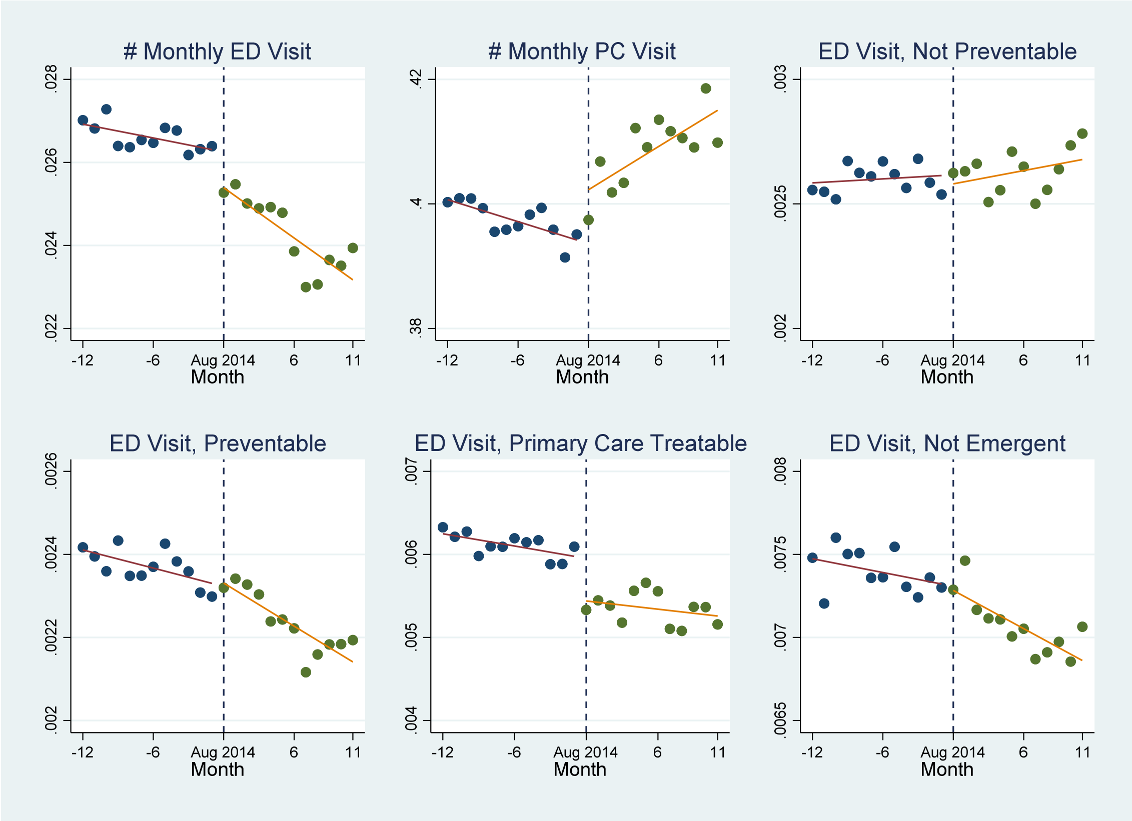

eFigure 2. Changes in monthly utilization rates before and after implementation (August 2014) of paid sick leave mandate among adult (18-64 years old) Medicaid beneficiaries in New York City.

Abbreviations: ED, emergency department; PC, primary care clinician.

Study sample included continuously-enrolled Medicaid beneficiaries aged 18–64 in New York City between August 2013–July 2015. Dots represent regression discontinuity coefficient estimates (see eAppenidx2 for details). Regression coefficients and 95% confidence interval were shown in eTable 5. Standard errors were clustered at the individual level.

© 2021 Glied SA et al. *JAMA Health Forum.*

eTable 1. Associations of the 2014 New York City paid sick mandate with annual health care use: event study

	Adjusted estimate (95% CI)					
	Any annual ED visit	# Annual ED visit	Any annual specialist visit	# Annual specialist visit	Any annual primary care clinician visit	# Annual primary care clinician visit
3 years before implementation X New York City	0.00001 (-0.002 to 0.002)	-0.00004 (-0.007 to 0.007)	-0.00003 (-0.004 to 0.004)	-0.0006 (-0.071 to 0.069)	0.0002 (-0.003 to 0.003)	0.0004 (-0.148 to 0.149)
2 years before implementation X New York City	0.00004 (-0.002 to 0.002)	-0.0001 (-0.008 to 0.007)	0.00008 (-0.004 to 0.004)	-0.0001 (-0.075 to 0.074)	0.0001 (-0.003 to 0.003)	0.001 (-0.150 to 0.153)
1 year before implementation X New York City (omitted)	–	–	–	–	–	–
Year of implementation X New York City	-0.006 (-0.008 to -0.004)	-0.024 (-0.032 to -0.016)	-0.010 (-0.014 to -0.006)	0.019 (-0.053 to 0.091)	0.002 (-0.001 to 0.005)	0.164 (0.034 to 0.293)
1 year after implementation X New York City	-0.006 (-0.008 to -0.004)	-0.018 (-0.026 to -0.011)	-0.010 (-0.014 to -0.006)	0.006 (-0.064 to 0.076)	-0.001 (-0.005 to 0.002)	0.081 (-0.053 to 0.216)
2 years after implementation X New York City	-0.007 (-0.009 to -0.005)	-0.023 (-0.030 to -0.015)	-0.012 (-0.016 to -0.008)	-0.089 (-0.161 to -0.017)	0.0003 (-0.003 to 0.004)	0.129 (-0.018 to 0.276)
Pre-policy outcome mean	0.496	0.874	0.605	3.954	0.784	4.902

Observations=3,317,142 person-years (N=552,857 persons).
Abbreviations: ED, emergency department.
Results were taken from regressions of outcome measures on the interactions between New York City indicator and year dummies (omitted category; the year prior to the implementation (August 2013–July 2014)). Regressions used sample reweighted by entropy balancing and include individual fixed effects, year dummies, and Charlson comorbidity index. Standard errors were clustered at the individual level.

© 2021 Glied SA et al. *JAMA Health Forum.*

eTable 2. Associations of the 2014 New York City paid sick mandate with emergent care utilization by type of visit: event study.

	Adjusted estimate (95% CI)			
	Emergent, not preventable	Emergent, preventable	Primary care treatable	Non-emergent
3 years before implementation X New York City	-0.000004 (-0.002 to 0.002)	-0.00001 (-0.001 to 0.001)	-0.00001 (-0.003 to 0.002)	-0.00002 (-0.003 to 0.003)
2 years before implementation X New York City	0.00004 (-0.002 to 0.002)	0.00002 (-0.001 to 0.002)	0.00006 (-0.002 to 0.002)	0.00005 (-0.003 to 0.003)
1 year before implementation X New York City (omitted)	–	–	–	–
Year of implementation X New York City	-0.0001 (-0.002 to 0.002)	-0.0008 (-0.002 to 0.001)	-0.003 (-0.005 to -0.0002)	-0.002 (-0.004 to 0.001)
1 year after implementation X New York City	0.0009 (-0.001 to 0.003)	-0.0008 (-0.002 to 0.0006)	-0.002 (-0.004 to 0.0003)	-0.001 (-0.004 to 0.001)
2 years after implementation X New York City	0.0007 (-0.001 to 0.003)	-0.0006 (-0.002 to 0.0009)	-0.003 (-0.005 to -0.0004)	-0.0004 (-0.003 to 0.002)
Pre-policy outcome mean	0.110	0.059	0.211	0.243

Observations=3,317,142 person-years (N=552,857 persons).
We classified emergency room use by type of visit by using Billings et al.'s algorithm that assigns likelihoods to each visit being each type on the basis of primary diagnosis (see eAppendix1 for details). Results were taken from regressions of outcome measures on the interactions between New York City indicator and year dummies (omitted category; the year prior to the implementation (August 2013–July 2014)). Regressions used sample reweighted by entropy balancing and included individual fixed effects, year dummies, and Charlson comorbidity index. Standard errors were clustered at the individual level.

© 2021 Glied SA et al. *JAMA Health Forum.*

eTable 3. Associations of the 2014 New York City paid sick mandate with adults' (40-64 years old) preventive care utilization: event study.

	Adjusted estimate (95% CI)				
	HbA1c test	Cholesterol test	Colon cancer screening	Breast cancer screening[a]	Cervical cancer screening[a]
3 years before implementation X New York City	0.00001 (-0.005 to 0.005)	0.0001 (-0.004 to 0.005)	-0.0001 (-0.005 to 0.005)	-0.004 (-0.011 to 0.004)	-0.002 (-0.009 to 0.005)
2 years before implementation X New York City	-0.00001 (-0.005 to 0.005)	0.00003 (-0.004 to 0.005)	-0.0001 (-0.005 to 0.005)	-0.0003 (-0.008 to 0.008)	-0.002 (-0.009 to 0.006)
1 year before implementation X New York City (omitted)	–	–	–	–	–
Year of implementation X New York City	0.021 (0.016 to 0.026)	0.026 (0.021 to 0.031)	0.003 (-0.002 to 0.008)	-0.008 (-0.015 to 0.0001)	-0.001 (-0.09 to 0.006)
1 year after implementation X New York City	0.021 (0.016 to 0.026)	0.024 (0.020 to 0.029)	0.007 (0.002 to 0.012)	-0.004 (-0.012 to 0.004)	0.003 (-0.005 to 0.010)
2 years after implementation X New York City	0.019 (0.014 to 0.024)	0.023 (0.019 to 0.028)	0.004 (-0.001 to 0.009)	-0.004 (-0.012 to 0.004)	-0.004 (-0.011 to 0.003)
Pre-policy outcome mean	0.549	0.741	0.165	0.525	0.259

Observations=1,917,156 person-years (N=319,428 persons).
[a] Among female Medicaid beneficiaries (N=194,186 persons).
Outcomes measured the probability that a person received a certain preventive service within a given year. Results were taken from regressions of outcome measures on the interactions between New York City indicator and year dummies (omitted category; the year prior to the implementation (August 2013–July 2014)). Regressions used sample reweighted by entropy balancing and included individual fixed effects, year dummies, and Charlson comorbidity index. Standard errors were clustered at the individual level.

© 2021 Glied SA et al. *JAMA Health Forum.*

eTable 4. Associations of the 2014 New York City paid sick mandate with annual health care utilization: alternative specification.

	Adjusted estimate (95% CI)					
	Any annual primary care clinician visit,	# Annual primary care clinician visit	Any annual specialist visit	# Annual specialist visit	Any annual ED visit	# Annual ED visit
Panel A. DID with entropy balancing weights (main specification)						
Post X New York City	0.0002 (-0.002 to 0.004)	0.124 (0.040 to 0.208)	-0.011 (-0.013 to -0.009)	-0.021 (-0.062 to 0.020)	-0.006 (-0.007 to -0.005)	-0.022 (-0.026 to -0.018)
Panel B. DID with Nearest neighbor propensity score matching						
Post X New York City	-0.001 (-0.003 to 0.001)	0.092 (0.018 to 0.166)	-0.014 (-0.016 to -0.012)	-0.027 (-0.062 to 0.008)	-0.006 (-0.014 to 0.002)	-0.014 (-0.032 to 0.004)
Panel C. DID without matching						
Post X New York City	0.0008 (-0.0008 to 0.002)	0.112 (0.020 to 0.204)	-0.005 (-0.011 to 0.001)	-0.020 (-0.055 to 0.015)	-0.005 (-0.009 to -0.001)	-0.012 (-0.028 to 0.004)
Panel D. Adding county-specific linear trends to the main specification						
Post X New York City	0.002 (-0.002 to 0.006)	0.150 (-0.013 to 0.313)	-0.009 (-0.013 to -0.005)	-0.029 (-0.076 to 0.018)	-0.005 (-0.007 to -0.003)	-0.023 (-0.033 to -0.013)
Panel E. Placebo regression#						
Post X New York City	-0.0002 (-0.002 to 0.002)	-0.0002 (-0.124 to 0.123)	-0.00008 (-0.004 to 0.004)	0.0005 (-0.062 to 0.063)	-0.00001 (-0.002 to 0.002)	0.0002 (-0.006 to 0.006)
Pre-policy outcome mean	0.784	4.902	0.605	3.954	0.496	0.874

Observations=3,317,142 person-years (N=552,857 persons).

It performed a regression analysis pretending that the policy took into effect a year earlier in New York City. This analysis included data only in the pre-policy period (observations=1,654,979).

Abbreviations: ED, emergency department; DID, difference-in-differences.

Regressions included year dummies, individual fixed effects, and Charlson comorbidity index. Standard errors were clustered at the individual level.

© 2021 Glied SA et al. *JAMA Health Forum.*

eTable 5. Changes in monthly utilization rates: regression discontinuity design.

	# monthly ED visit	# monthly primary care clinician visit	ED visit –Not preventable	ED visit – Preventable	ED visit – Primary care treatable	ED visit – Not emergent
Discrete change in utilization rate	-0.002 (-0.001 to -0.002)	0.013 (0.010 to 0.015)	-0.0003 (-0.002 to 0.001)	0.0002 (-0.0001 to 0.0003)	-0.0004 (-0.001 to -0.0003)	-0.0005 (-0.0013 to 0.0003)
Bonferroni-corrected P value	<0.001	<0.001	0.623	0.756	<0.001	0.503
Change in temporal trend	0.0001 (-0.00003 to 0.0003)	0.002 (0.002 to 0.003)	0.0001 (0.00002)	-0.00001 (-0.00003 to 0.00003)	0.00003 (-0.00001 to 0.0001)	-0.000001 (-0.00006 to 0.00006)
Bonferroni-corrected P value	0.502	<0.001	<0.001	0.732	0.368	0.908
Pre-policy outcome mean	0.027	0.393	0.0025	0.0024	0.006	0.007

Observations=8,668,992 person-month (N=361,208 persons).
Abbreviations: ED, emergency department.
Study sample included continuously-enrolled Medicaid beneficiaries aged 18–64 in New York City between August 2013–July 2015. Regressions also included month dummies (February through December) and individual fixed effects. Standard errors were clustered at the individual level. Detailed information on the regression framework was provided in eAppendix2.

© 2021 Glied SA et al. *JAMA Health Forum.*
